# Single GDP-dissociation Inhibitor Protein regulates endocytic and secretory pathways in *Leishmania*

**DOI:** 10.1038/srep37058

**Published:** 2016-11-14

**Authors:** Senthil kumar Shanmugam, Kamal Kumar, Pawan Kishor Singh, Ruchir Rastogi, Amitabha Mukhopadhyay

**Affiliations:** 1 National Institute of Immunology, Aruna Asaf Ali Marg, New Delhi 110067, India

## Abstract

The role of GDP dissociation inhibitor (GDI) protein in regulation of Rab cycle in *Leishmania* is not known. Here, we have cloned and characterized the functions of GDI homologue *in vivo* in *Leishmania*. Our results have shown that LdGDI:WT along with GDP removes the Rab5 from purified endosomes and inhibits the homotypic fusion between early endosomes. Whereas, LdGDI:R239A, a dominant negative mutant of GDI, under the same condition neither removes the Rab5 from endosome nor inhibits fusion. To determine the role of Ld-GDI *in vivo*, transgenic parasites overexpressing GFP-LdGDI:WT or GFP-LdGDI:R239A, are co-expressed with RFP-LdRab5:WT, RFP-LdRab7:WT or RFP-LdRab1:WT. Our results have shown that overexpression of GFP-LdGDI:WT extracts the RFP-LdRab5, RFP-LdRab7 or RFP-LdRab1 from their discrete endomembrane predominantly into cytosol. No change in the distribution of indicated Rabs is detected with overexpression of GFP-LdGDI:R239A. To determine the functional significance, we have used hemoglobin as an endocytic marker and gp63 as a marker for secretory pathway. We have found that overexpression of GFP-LdGDI:WT enhances the lysosomal targeting of internalized hemoglobin and the secretion of gp63 in the parasites possibly by triggering Rab cycle. This is the first demonstration of a single GDI ubiquitously regulating both endocytic and secretory pathways in *Leishmania*.

Rab proteins are the largest family of small GTPases of the Ras superfamily which are master regulators of intracellular trafficking^1^,^2^. They are present in all eukaryotes ranging from protozoan parasites to mammalian cells^3^. *In vitro* studies in mammalian cells have shown that Rab proteins oscillate between the inactive GDP-bound conformation and the active GTP-bound state and thereby act as molecular switches to regulate intracellular transport^4^,^5^. Similarly, Rabs are also cycled between cytosol and membrane and Rab-GDI plays a central role in the recycling of the Rab proteins^5^. The newly synthesized Rabs require the Rab escort protein (REP) for recognition by Rab geranylgeranyl transferase (RGGT)^6^,^7^. Subsequently, REP delivers the prenylated Rab to the target membrane^8^,^9^ along with Rab-GDI. Membrane bound Rabs are activated by a specific guanine nucleotide-exchange factor (GEF) and subsequently GTP-form of the Rab interacts with various downstream effectors to execute their function^10^. Following membrane fusion, Rab proteins are deactivated by a GTPase-activating protein (GAP) which hydrolyzes GTP to GDP^11^,^12^. Rab-GDI preferentially binds with GDP-bound conformation of the Rab and extracts it from the membrane into cytosol with the help of GDI displacement factor^13^,^14^. Finally, Rab cycle is completed when GDP-bound Rab is presented to the new membrane along with REP^15^. Thus, GDI controls the distribution of Rabs between membranes and cytosol and plays critical role for the proper functioning of the vesicular transport machinery.

*Leishmania donovani*, a pathogenic parasite, causes visceral leishmaniasis, a fatal human disease that affects annually about 12 million people worldwide^16^,^17^. Both endocytic and secretory trafficking pathways regulated by various Rab GTPases are well conserved in Trypanosomatid parasites^18^,^19^. We have also characterized role of Rab5 and Rab7 in the endocytic pathway of *Leishmania*^20^,^21^,^22^ using hemoglobin endocytosis in the parasite^23^,^24^,^25^,^26^. Similarly, we have found that Rab1 regulates the conventional secretory pathway in *Leishmania*^27^. Thus, this unicellular parasite offers a great model to study the intracellular trafficking pathways *in vivo* in an organism. In the present investigation, we aim to understand the regulation of Rab cycle by GDI in *Leishmania*, which is not yet characterized. Nonetheless, the presence of Rab-GDI homologue in *Plasmodium falciparum* and *Giardia lamblia* is reported^28^,^29^, but the functional role of Rab-GDI in regulating Rab cycle in any of these parasite is not yet elucidated. Here, we report the cloning, expression and functional characterization of Rab-GDI homologue from *Leishmania donovani*. We have shown that single LdGDI regulates the distribution of cytosolic and membrane bound Rabs both in endocytic and secretory pathways and thereby regulates intracellular trafficking in *Leishmania*.

## Results

### Cloning and expression of GDI homologue from *Leishmania donovani*

**Figure 1:**
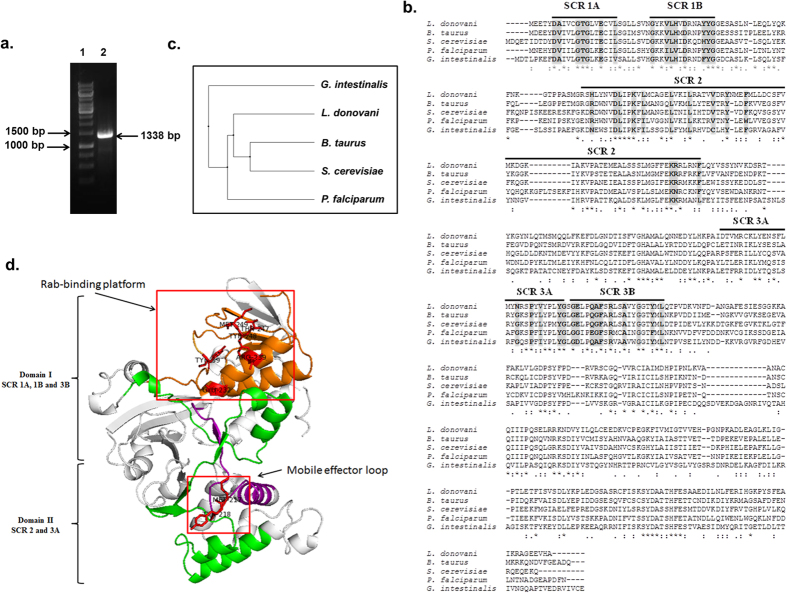
Cloning of LdGDI from *Leishmania donovani*. (**a**) 1338 bp fragment (lane 2) was amplified from *L. donovani* cDNA by PCR using appropriate forward and reverse primers as described in Methods. Lane 1 represents DNA ladder. The sequence of LdGDI was submitted to GenBank database under accession no. KX181889. (**b**) Hypothetical translation of the obtained sequence into protein revealed that LdGDI shows significant similarity with Rab-GDI from *B. Taurus, S. cerevisiae, P. falciparum* and *G. intestinalis*. Residues corresponding to 5 characteristic conserved or SCR of Rab-GDI are marked with line and the consensus sequences in SCRs of GDI are indicated in Bold. (**c**) Phylogenetic comparison of LdGDI with GDI from different organisms. The dendrogram was generated by Jalview program based on percent identity. (**d**) Putative 3D structure of LdGDI based on homology-modeling using SWISS-MODEL automated protein structure server. SCR 1 and 3B are shown in orange, SCR 3A in purple and SCR 2 in green colour. Residues found in Rab-binding platform and mobile effector loop are shown in red sticks.

To clone a GDI homologue from *L. donovani*, we amplified a 1338 bp fragment from *L. donovani* cDNA by PCR using appropriate forward and reverse primers (Fig. 1a). The PCR product was cloned, sequenced and hypothetically translated into 445 amino acid sequence. The obtained sequence was analyzed by multiple protein sequence alignment using Clustal Omega^30^ which demonstrated that LdGDI has about 51% sequence identity with *B. taurus* GDI, 47% with *S. cerevisiae* GDI, 43% with *P. falciparum* GDI and 39% with *G. intestinalis* GDI (Fig. 1b). Subsequently, GST-LdGDI:WT fusion protein of expected size (~75 kDa) was purified to homogeneity (Supplementary Fig. 1). Similarly, cells transformed with mutant plasmid were used to purify the GST-LdGDI:R239A fusion protein (data not shown). The sequence analysis of LdGDI also revealed the presence of 5 characteristic Rab-GDI conserved region or SCR, like SCR 1A, SCR 1B, SCR 2, SCR 3A and SCR 3B in the amino terminal and the central region of the protein. The alignment result was also analyzed using Jalview program^31^ and a dendrogram was generated using percent identity to find the phylogenetic relationship of LdGDI with its different orthologues. The dendrogram result suggested the early divergence of *Leishmania* from another protozoan parasite *G. intestinalis* and its close association with mammalian (*B. taurus*) or yeast (*S. cerevisiae*) GDI (Fig. 1c).

To appreciate the putative structure of LdGDI, we modeled the LdGDI protein sequence based on *B. taurus* GDI structure (PDB ID 1D5T) as it showed maximum identity. We used SWISS-MODEL fully automated protein structure homology-modelling server for modeling LdGDI^32^. The obtained model was analyzed using pymol molecular visualization system. Our modeling showed (Fig. 1d) that like bovine α-GDI structure, LdGDI also has two domains, a larger domain I composed of mainly β-sheets and a smaller domain II composed of α-helix. We also found that domain I is composed of SCR 1A, SCR 1B and SCR 3B, which forms the Rab-binding platform at the apex of LdGDI where Rab-GDI interacts with the switch region of Rab GTPases^33^. Whereas, domain II containing mobile effector loop encompassing SCR 3A was found to be present at the base of the protein, that was shown to be involved in membrane interaction. SCR 2 was found to be connecting both domains of LdGDI^34^. In bovine α-GDI, residues Tyr 39, Glu 233, Arg 240, Thr 248, Tyr 249, and Met 250 were predicted to play crucial roles in the binding to Rabs^35^. Our results showed that these residues are corresponding to Tyr 39, Glu 232 Arg 239, Thr 247, Tyr 248, and Met 249 in LdGDI, which form part of the SCR1 (Tyr 39) and SCR3B (Glu 233, Arg 240, Thr 248, Tyr 249, and Met 250), respectively (Fig. 1b).

### Localization of LdGDI in *Leishmania*

**Figure 2:**
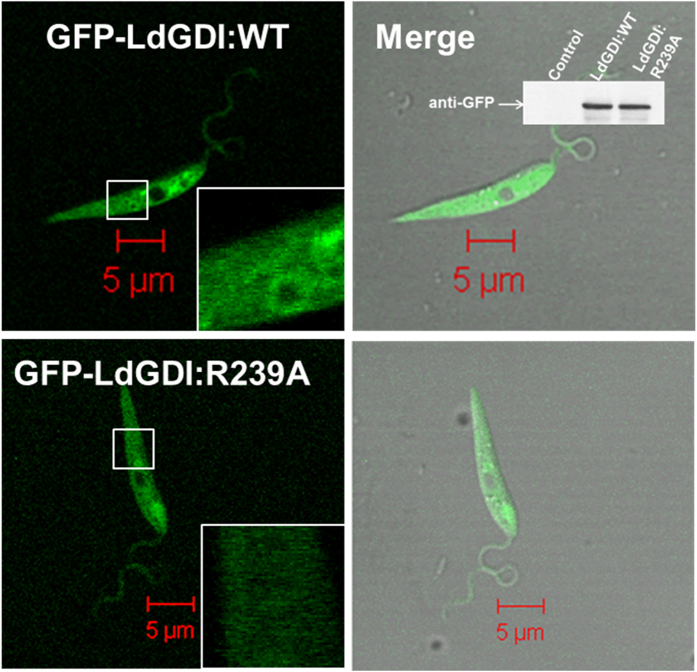
Localization of LdGDI:WT and its mutant in *Leishmania*. To determine the localization of LdGDI:WT and its mutant in *Leishmania*, cells were transfected with indicated constructs to overexpress the respective protein in *Leishmania* as GFP fusion protein. Cells were visualized in LSM 510 Meta confocal microscope. Inset shows the levels of overexpression of LdGDI:WT and its mutant as GFP fusion proteins in *Leishmania*. Cell lysates were analyzed by western blotting using anti-GFP antibody. Untransfected *Leishmania* was used as control. Full length blot is presented in Supplementary Fig. 2. Results are representative of three independent preparations.

LdGDI or its mutant was overexpressed in *Leishmania*, as GFP fusion proteins using pXG-GFP^+2^ *Leishmania* expression vector to determine their localization. Stable clones were selected in the presence of G418 antibiotic and the overexpression of respective protein as GFP fusion protein in *Leishmania* was confirmed by western blot analysis of the cell lysates using anti-GFP antibody as well as by confocal microscopy. GFP-LdGDI:WT was found to be distributed both in cytosol and membranous structures in *Leishmania*, whereas GFP-LdGDI:R239A predominantly localized in the cell cytosol (Fig. 2). Western blot analysis with anti-GFP antibody showed the comparable expression of GFP-LdGDI:WT and GFP-LdGDI:R239A in *Leishmania* (Fig. 2, inset).

### Functional characterization of LdGDI from *Leishmania*

**Figure 3:**
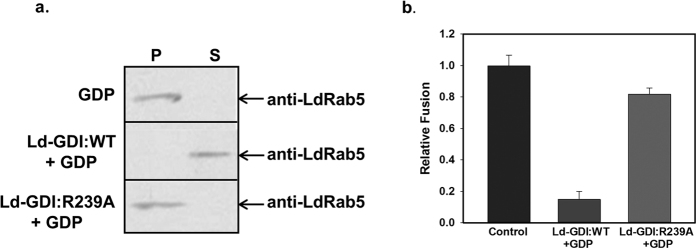
Functional characterization of LdGDI. (**a**) Extraction of LdRab5 from the endosome by LdGDI. Endosome treated with GDP alone was used as a control. Treated endosomes were centrifuged and pellets were analyzed for the presence of LdRab5 by western blots using anti-LdRab5 antibody. Full length blot is presented in Supplementary Fig. 3. Results are representative of three independent preparations. (**b**) Role of LdGDI in *in vitro* homotypic fusion between endosomes in *Leishmania*. To determine the role of LdGDI, *in vitro* homotypic fusion between endosomes containing BHb and endosome containing Avidin-HRP (AHRP) was carried out as described in Methods. LdGDI or its mutant treated and untreated (control) endosomes containing BHb was used in the fusion assay. Fusion obtained with untreated endosomes was chosen as 1unit and results are expressed as relative fusion of three independent experiments ± S.D. One unit corresponds to 12.8 ng of HRP activity/mg of protein.

It is well evident that dominant negative mutant protein is very useful to determine the function of the respective wild type protein in intracellular transport processes. Previous studies have shown that substitution of arginine (R) at position 240 to alanine (A) in SCR3B of the mammalian GDI impairs its function^35^. Accordingly, LdGDI:R239A mutant was generated by substituting arginine at position 239 to alanine by site-directed mutagenesis of SCR3B of LdGDI. To characterize the cloned protein as a functional GDI in *Leishmania*, we exploited the properties of Rab-GDI to extract the Rab proteins from the vesicle membrane in the presence of GDP as demonstrated in mammalian cells^36^. Therefore, LdRab5-positive purified early endosome containing biotinylated-hemoglobin (BHb) was incubated with LdGDI:WT, or LdGDI:R239A in the presence or absence of 1 mM GDP. Subsequently, distribution of LdRab5 on endosomal membranes and cytosol was determined by western blot analysis using a specific antibody. The results presented in the Fig. 3a showed that LdGDI along with GDP removes LdRab5 from the endosomal membrane to the cytosol whereas GDP alone was unable to remove LdRab5 from the membrane. Interestingly, treatment of endosomal membrane with LdGDI:R239A along with GDP was unable to remove the LdRab5 from the membrane indicating that LdGDI:R239A functions as a dominant negative mutant of LdGDI.

To ascertain if wild type and mutant proteins is a functional GDI in *Leishmania, in vitro* homotypic fusion between endosomes containing BHb with endosome containing Avidin-HRP (AHRP) was carried out. To determine the role of LdGDI, one set of endosome containing BHb was treated with LdGDI:WT or LdGDI:R239A in the presence of GDP prior to the fusion assay. Untreated endosomes were used as a control. Our results showed that BHb containing endosomes treated with LdGDI in presence of GDP inhibited about 80% fusion with endosomes containing AHRP in comparison to control (Fig. 3b). Whereas, only about 15% inhibition of fusion among these endosomes was observed when BHb containing endosomes were treated with LdGDI:R239A along with GDP. Taken together, these results demonstrated that LdGDI from *Leishmania* is a functional GDI.

### Extraction of Rab GTPases from the endomembrane in *Leishmania*

**Figure 4:**
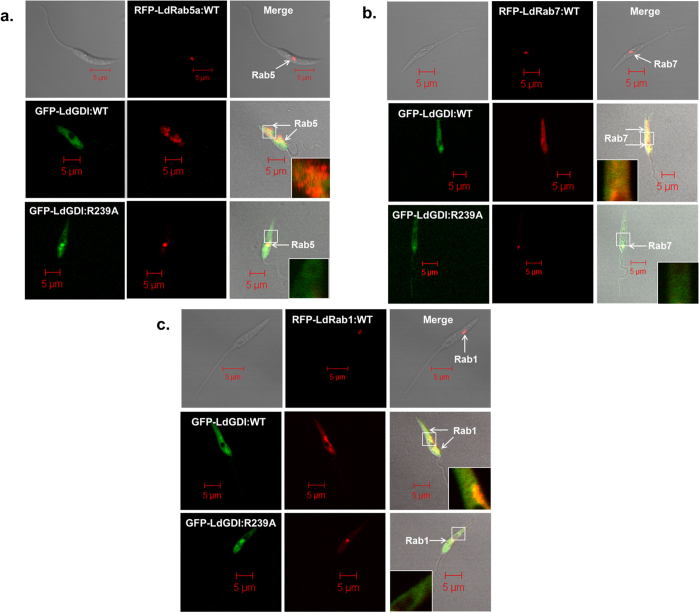
Extraction of various Rab GTPases by LdGDI from the endomembrane in *Leishmania*. To determine the role of LdGDI *in vivo*, GFP-LdGDI:WT and GFP-LdGDI:R239A expressing cells were co-expressed with RFP-LdRab5:WT, RFP-LdRab7:WT or RFP-LdRab1:WT and compared the distribution of indicated Rabs in *Leishmania* by confocal microscopy as described in Methods. (**a**) Co-expression of GFP-LdGDI:WT or GFP-LdGDI:R239A with RFP-LdRab5:WT. (**b**) Co-expression of GFP-LdGDI:WT or GFP-LdGDI:R239A with RFP-LdRab7:WT. (**c**) Co-expression of GFP-LdGDI:WT or GFP-LdGDI:R239A with RFP-LdRab1:WT. Results are representative of three independent preparations.

To determine the role of LdGDI *in vivo*, GFP-LdGDI:WT and GFP-LdGDI:R239A expressing cells were co-expressed with RFP-LdRab5:WT, RFP-LdRab7:WT or RFP-LdRab1:WT and compared the distribution of indicated Rabs in *Leishmania*. Consistent with our previous finding, RFP-LdRab5:WT localized predominantly in discrete early endosomal compartment^20^ in more than 95% of control cells, whereas RFP-LdRab5:WT was found to be redistributed in the cytosol in more than 90% of GFP-LdGDI:WT coexpressing *Leishmania*. No change in the distribution of RFP-LdRab5:WT was detected in GFP-LdGDI:R239A expressing cells (Fig. 4a). Similarly, RFP-LdRab7 was extracted into *Leishmania* cytosol from their respective compartment^21^ in more than 95% of GFP-LdGDI:WT coexpressing *Leishmania* (Fig. 4b). In addition, we also found that expression of GFP-LdGDI:WT in *Leishmania* removes the RFP-LdRab1:WT from their discrete localization in Golgi^27^ to cytosol in more than 95% of cells (Fig. 4c). In contrast, GFP-LdGDI:R239A expressing cells were unable to extract either RFP-LdRab7:WT or RFP-LdRab1:WT proteins into cytosol. Interestingly, indicated LdRabs were also found to be present on appropriate membrane in GFP-LdGDI:WT expressing cells as GDI was also shown to present extracted Rabs to the donor membrane^15^. Thus, overexpression of LdGDI:WT not only extracted the indicated Rabs from the respective endomembrane, but also presented them to the appropriate membrane indicating typical functions of established Rab-GDI.

### Role of LdGDI in endocytic and secretory pathways in *Leishmania*

**Figure 5:**
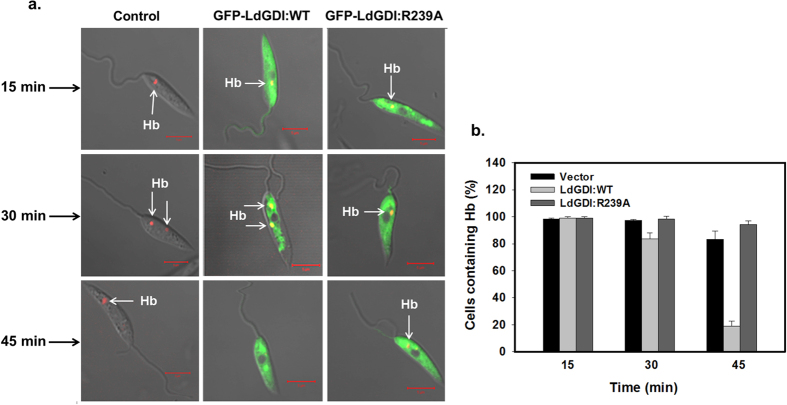
Role of LdGDI in endocytic pathway in *Leishmania*. (**a**) To determine the role of LdGDI in endocytic pathway of *Leishmania* promastigotes, we analyzed the trafficking of Alexa Fluor 594-conjugated Hb in the cells overexpressing GFP-LdGDI:WT or GFP-LdGDI:R239A using confocal microscopy as described in Methods. Untransfected cells were used as a control. Green, GFP-LdGDI:WT or GFP-LdGDI:R239A; Red, Alexa Fluor 594-conjugated Hb. Results are representative of three independent preparations. (**b**) Quantitative analysis of percentage of cells showing the presence of Alexa-594-Hb at indicated time points of internalization in different cell types. Results are expressed as mean ± SD.

Previously, we showed that *Leishmania* internalizes Hb via a clathrin-dependent receptor-mediated endocytic process^23^,^25^. To determine the role of LdGDI in endocytic pathway of *Leishmania* promastigotes, we followed the trafficking of Alexa Fluor 594-conjugated Hb in the respective cells by confocal microscopy. The results presented in Fig. 5a (right panel) showed that bound Hb internalized into an early endosomal compartment by 15 min, which is subsequently transported to the perinuclear late compartment by 30 min and finally reached lysosomal compartment by about 45 min in control cells as shown previously^21^. In contrast, Hb was rapidly internalized into early endocytic compartment and targeted to the late endosome/lysosomal compartments within 30 min in cells overexpressing GFP-LdGDI:WT (Fig. 5a, middle panel), and almost no Hb was found in these cells after 45 min indicating complete degradation of internalized Hb. In contrast, Hb trafficking to the late compartment was inhibited in GFP-LdGDI:R239A expressing cells in comparison to untransfected control cells (Fig. 5a, left panel). Further quantitative analysis of several micrographs (n = 50) revealed (Fig. 5b) that more than 80% of the GFP-LdGDI:WT expressed parasites has no Hb after 45 min of internalization, whereas almost all control and GFP-LdGDI:R239A expressing parasites contain Hb.

**Figure 6:**
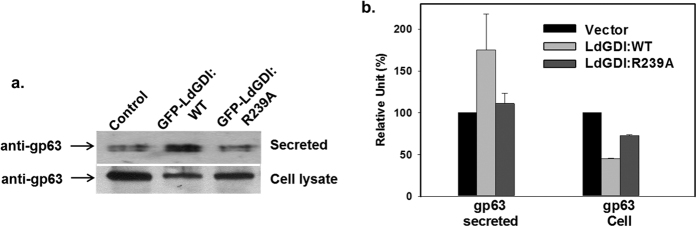
Role of LdGDI in the secretory pathway in *Leishmania*. (**a**) To determine the role of LdGDI in the secretory pathway in *Leishmania*, we determined the levels of cell-associated and secreted gp63 by respective *L. donovani* promastigotes by western blot analysis using specific antibodies as described in Methods. Full length blot is presented in Supplementary Fig. 4. Results are representative of three independent experiments. (**b**) Quantitative analysis of amount of gp63 present in the cell lysate and secreted by indicated parasites. Amount of gp63 present in the cells and secreted by vector control cells were taken as 100 Unit. Results are expressed as mean ± SD.

GP63 is the major cell surface associated GPI-anchored protein in *Leishmania*, which is spontaneously secreted by the cells^37^. Therefore, to determine the role of LdGDI in the secretory pathway, we measured the amount of endogenous gp63 secreted into *Leishmania* culture medium by GFP-LdGDI:WT or GFP-LdGDI:R239A overexpressing cells after 24 h at 23 °C. The amount of gp63 secreted by untransfected cells was used as a control. We found that GFP-LdGDI:WT overexpressed parasites secreted significantly higher amounts of gp63 than untransfected control cells (Fig. 6a). In contrast, no significant difference in the secretion of gp63 was observed in GFP-LdGDI:R239A overexpressed cells in comparison to control as it can’t bind Rabs. Enhanced secretion of gp63 by GFP-LdGDI:WT overexpressed parasites was supported by the significantly less amount of intracellular content of gp63 in this cells in comparison to control cells (Fig. 6a). Further quantitation revealed that LdGDI:WT overexpressed parasites secrete about 50% higher amount of gp63 than untransfected control cells (Fig. 6b). Similarly, intracellular content of gp63 was found to be significantly less in LdGDI:WT overexpressed parasites in comparison to control and LdGDI:R239A overexpressed cells.

## Discussion

Previously, we have shown that Rab5 and Rab7 regulate the endocytic pathway in *Leishmania*, whereas the conventional secretory pathway in the parasites is regulated by its Rab1 homologue^20^,^21^,^27^. We have also shown that GTP form of the respective Rabs are present on appropriate membrane in *Leishmania*, whereas inactive GDP form of the parasites Rabs are predominantly present in parasite cytosol. But, how these proteins move between membrane and cytosol is not known in parasites. It has been shown in mammalian cells that Rab-GDI plays a central role in the recycling of Rab proteins between cytosol and membrane^5^ using mainly an *in vitro* stripping assay. As several components of intracellular trafficking machinery are well conserved in *Leishmania*, we have cloned and expressed LdGDI homologue from *Leishmania* to determine the role of GDI in regulating intracellular trafficking pathways *in vivo* in this parasite.

We have amplified a 1338 bp fragment from *L. donovani* cDNA by PCR which codes for ~50 kDa protein. Sequence analysis of the cloned protein reveals the presence of 5 characteristic Rab-GDI conserved region or SCR, like SCR 1 A, SCR 1B, SCR 2, SCR 3 A and SCR 3B in the amino terminal and the central region of protein. The consensus sequences of SCRs of GDI^38^ is reported as: DVxxxGTGxxExxL (SCR 1 A), GxxVLHxD xxxYYG (SCR1B) and GExxQGFx RxxAxxG (SCR 3B). Interestingly, we have observed two changes in the conserved motifs of SCR1 and SCR3B in LdGDI; i. alanine in the position of valine in SCR 1 A (D**A**xxxGTGxxExxL) and ii. alanine in the position of Glycine in SCR 3B (GExxQ**A**FxRxxAxxG). Presence of these motifs in SCRs on the surface of LdGDI suggests parasite-specific roles of LdGDI in intracellular trafficking in *Leishmania*. Moreover, LdGDI shows high degree of homology with GDI reported from different organisms and maximum identity is observed with *B. taurus* GDI. In addition, dendrogram results have also shown that *Leishmania* has close association with mammalian (*B. taurus*), or yeast (*S. cerevisiae*) GDI than *G. intestinalis* GDI. These results indicate that LdGDI is a GDI homologue from *Leishmania*.

A large number of studies have shown that the appropriate mutation within a conserved region of a transport related protein renders the protein either constitutively active or dominant negative mutants that are very useful tools to determine the function of the endogenous protein in intracellular transport^39^,^40^. Therefore, to determine the function of LdGDI in trafficking in *Leishmania*, we have generated a LdGDI:R239A mutant by substituting arginine at position 239 to alanine. Since GDI is known to remove Rab proteins from the vesicular membrane^36^,^41^,^42^, we have purified LdRab5-positive early endosomes containing biotinylated-hemoglobin (BHb) from *Leishmania*^20^ and added with GST-LdGDI:WT or GST-LdGDI:R239A in the presence or absence of 1 mM GDP. Our results have shown that GST-LdGDI:WT selectively removes the LdRab5 from endosomes. In addition, it has been shown that removal of Rab proteins from vesicle membranes by Rab-GDI treatment inhibits the function of Rabs. For example, Rab5 stripped from phagosomes by GDI:WT protein inhibits the fusion with endosomes^42^. Similarly, we have found that LdGDI treated BHb-containing endosomes are unable to fuse with AHRP-containing endosomes in an *in vitro* fusion assay. These results demonstrate that LdGDI is a functional homologue of the GDI in *Leishmania*. Moreover, treatment of endosomes with GST-LdGDI:R239A under similar condition neither removes LdRab5 from endosomes, nor inhibits fusion indicating that the mutant protein is dominant negative mutant of LdGDI.

Even though the function of Rab-GDI in mammalian cells is well characterized *in vitro*^36^, however, its role *in vivo* is not very well depicted. Therefore, we have co-expressed GFP-LdGDI:WT or GFP-LdGDI:R239A along with RFP-LdRab5:WT, RFP-LdRab7:WT or RFP-LdRab1:WT in *Leishmania* to determine the function of LdGDI *in vivo*. Consistent with our previous finding, LdRab5, LdRab7 and LdRab1 are localized onto discrete early endosomes, late endosome and Golgi, respectively when overexpressed separately. As expected, these proteins are found to be redistributed into the parasite cytosol significantly more when GFP-LdGDI:WT is co-expressed in *Leishmania*. However, respective Rabs are also found to be present on the endomembrane of GFP-LdGDI:WT co-expressed in *Leishmania*, as Rab-GDI is known to present the GDP form of Rabs to donor membrane^15^. Though, some vertebrates and plants possess multiple isoforms of GDI^8^, our results have shown that LdGDI ubiquitously extracts both endocytic and secretory Rabs indicating the existence of single GDI in *Leishmania*. This is supported by the fact that only one GDI homologue is present in *Leishmania* genome database.

To analyze the role of LdGDI in endocytic pathway in *Leishmania*, we have studied the trafficking of endocytosed Hb in GFP-LdGDI:WT and GFP-LdGDI:R239A overexpressed cells. Previously, we have shown that *Leishmania* endocytosed Hb via a clathrin-dependent receptor-mediated endocytic process^23^,^25^. Consistent with this previous finding, we have found that bound Hb internalized into early endosomal compartment by 15 min, which is subsequently transported to the lysosomal compartment by about 45 min in control cells. Whereas, in GFP-LdGDI:WT overexpressed cells, Hb is rapidly internalized into early and late endocytic compartments and targeted to lysosomal compartments within 30 min, where it is degraded. It appears that overexpression LdGDI triggers the endocytic pathway in *Leishmania*, possibly by enhancing the Rab cycle by simultaneous promoting extractions and presentation of endocytic Rabs (e.g., LdRab5 and LdRab7) on appropriate membrane. Consequently, we have determined the role of LdGDI in the secretory pathway by measuring the amount of endogenous gp63 secreted into *Leishmania* culture medium by GFP-LdGDI:WT overexpressing cells. Previous studies have shown that gp63 is a cell surface associated GPI-anchored protein in *Leishmania*^43^, which is synthesized as an inactive precursor and targeted to the endoplasmic reticulum^44^. Finally, the majority of gp63 is secreted out of the cell^45^ by a LdRab1-dependent conventional secretory pathway^27^. Interestingly, our results have shown that GFP-LdGDI:WT overexpressed parasites secrete significantly higher amounts of gp63 than untransfected control cells. Enhanced secretion of gp63 by GFP-LdGDI:WT overexpressed parasites is supported by the significantly less amount of intracellular content of gp63 in these cells. Thus, like the endocytic pathway, LdGDI must have enhanced the LdRab1 cycle in parasites, which culminates in enhanced secretion of gp63 by parasites.

In conclusion, this is the first *in vivo* demonstration of the functional role of a Rab-GDI in regulating intracellular trafficking in *Leishmania*. We have shown that LdGDI ubiquitously extracted multiple Rabs consistent with the existence of single GDI in *Leishmania*. In addition, our results suggest that LdGDI not only extracts a Rab from its respective endomembranes, but also presents them to the donor membrane and thereby enhances the Rab cycle to facilitate intracellular trafficking in *Leishmania*.

## Methods

### Materials

Unless otherwise mentioned, all reagents were obtained from Sigma Chemical Co. (St. Louis, MO). M199 medium, Gentamicin and Geneticin were purchased from GIBCO-BRL (Gaithersburg, MD). Fetal Calf Serum (FCS) was procured from Biological Industries (Beit-Haemek, Israel). Platinum High Fidelity Taq polymerase and restriction enzymes were purchased from Invitrogen (Carlsbad, CA) and Promega Life Science (Madison, WI), respectively. pGEX-4T-2 expression vector, Glutathione Sepharose 4B beads, protein markers (RPN756 and RPN800) and ECL reagents were obtained from Amersham Bioscience (Amersham, UK). Site-Directed Mutagenesis Kit (QuikChange^®^) was purchased from Stratagene (La Jolla, USA). The *Leishmania* expression vectors, pXG-GFP^+2^ and pNUS-mRFP-nD were kindly provided by Dr. S. M. Beverley (Washington University, St. Louis, MO) and Dr. Jean-Paul di Rago (Institut de Biochimie et Génétique Cellulaires, Bordeaux, France) respectively. All other reagents used were of analytical grade.

### Cells

Promastigotes of *Leishmania donovani* (UR6) were obtained from Indian Institute of Chemical Biology, Kolkata, India. Cells were cultured in medium M199 (pH 7.4) supplemented with 10% FCS, 100 units/ml penicillin, 100 μg/ml streptomycin at 23 °C, and log-phase cells were harvested in phosphate-buffered (10 mM, pH 7.2) saline (0.15 M).

### Cloning of Rab-GDI from *Leishmania donovani*

To clone the GDI homologue from *Leishmania*, a putative GDI-like sequence was identified from *Leishmania major* genome database having substantial homology with GDI reported from different organisms by BLAST analysis. Accordingly, appropriate forward (5^′^-CGTGGATCCATGGAGGAGACGTACGATGCG-3^′^) and reverse primers (5^′^-CGT GAATTCCTACGCATGTACCTCCTCACC-3^′^) were designed against start and stop codons of putative *L. major* GDI sequence with BamHI and EcoRI restriction sites, respectively. The ORF of the putative GDI sequence was amplified from *L. donovani* cDNA using these primers by PCR. Briefly, mRNA isolated from *Leishmania* promastigotes using Oligotex mRNA kit (Qiagen) was used for cDNA synthesis using Thermo Script RT-PCR kit (GibcoBRL) as per manufacturer’s instruction. Subsequently, PCR was performed using above primers in a Perkin-Elmer thermocycler for 30 cycles as per the following conditions: denaturation for 30 sec min at 98 °C; annealing at 58 °C for 30 sec and extension at 68 °C for 2 min. A 1338 bp fragment amplified by PCR was cloned into pGEM-T Easy vector and sequenced by using M13 universal primers in an automated sequencer. Finally, the PCR product was cloned into BamHI/EcoRI sites of pGEX-4T-2 expression vector and transformed into XL-1 Blue strain of *Escherichia coli*. The pGEX-4T-2:LdGDI plasmid was transformed into BL-21 strain of *E. coli* for expression and purification of the GST-LdGDI fusion protein. Similarly, cells transformed with mutant plasmid were used to purify the GST-LdGDI:R239A fusion protein (data not shown).

### Generation of LdGDI mutants

To investigate the functions of LdGDI more precisely, LdGDI:R239A mutant was made by replacing Arginine^239^ with Alanine using the QuikChange^®^ Site-Directed Mutagenesis Kit according to the manufacturer’s protocol. For the constructing LdGDI:R239A, mutant primers (Forward: 5^′^-GCAGGCCTTCTCGGCCCTGTCGGCCG-3′ and Reverse: 5^′^-CGGCCGACAGG GCCGAGAAGGCCTGC-3^′^) complimentary to opposite strands of the LdGDI gene were designed according to the kit recommendation. Using these primers, the desired mutation (GCG codon changed to GGC) was introduced into pBluescript:LdGDI template by PCR. The resulting PCR product was digested with Dpn I, which cleaves the methylated template DNA. Subsequently, the nicked plasmid DNA containing the desired mutations was transformed into XL1-Blue supercompetent cells and the recombinant pBluescript:LdGDI:R239A recovered from the transformants by standard techniques. Finally, LdGDI:R239A was subcloned into BamHI/EcoRI sites of pGEX-4T-2 expression vector and transformed into XL-1 Blue strain of *Escherichia coli*.

### Overexpression of LdGDI and Its Mutant in *Leishmania*

To overexpress LdGDI and its mutant in *Leishmania* as a GFP fusion protein, respective clones were subcloned into the BamH1/EcoR1 sites of the pXG-GFP^+2^ vector^46^. Subsequently, *Leishmania* promastigotes were transfected with LdGDI:WT or its mutant constructs using previously described process^21^. Finally, positive clones were selected in the presence of G418 antibiotic (30 μg/ml). Overexpression of respective protein was confirmed by western blotting using anti-GFP antibody. Cells were visualized under confocal microscopy to determine the localization of LdGDI and its mutant in *Leishmania*.

### Preparation of BHb or avidin-HRP containing early endosomes from *Leishmania*

Biotinylated hemoglobin (BHb) containing early endosomes from *Leishmania* were purified as described previously^20^. Briefly, *Leishmania* promastigotes were incubated with BHb (2 mg/ml) in internalization medium (MEM containing 10 mM HEPES and 5 mM glucose, pH 7.4) for 5 min at 23 °C to label the early endosomal compartment. Internalization of BHb was stopped by the addition of cold medium and the cells were washed five times with PBS. Subsequently, cells were resuspended (5 × 10^9^ cells/12 ml) in homogenization buffer (HB, 20 mM Hepes, 250 mM sucrose and 2 mM EGTA, pH 7.2 containing protease inhibitors), equilibrated in a pre-cooled nitrogen cavitation vessel (Parr Instrument Company, IL) with 750 psi N_2_ for 25 min at 4 °C and disrupted by release of N_2_ from the bomb. The unbroken cells, nucleus and other cell debris were removed by low speed centrifugation at 500 g for 10 min at 4 °C. The post-nuclear supernatant (PNS) containing early endosomes was quickly frozen in liquid nitrogen. Subsequently, thawed PNS was diluted with HB (1:3) and centrifuged at 20000 g for 1 min at 4 °C. The resultant supernatant was again centrifuged at 100000 g for 5 min at 4 °C. The pellet enriched in early endosome containing respective probe was used for *in vitro* fusion assays. Similarly, another set of endosomes containing Avidin-HRP was prepared.

### Removal of LdRab5 from early endosomes by LdGDI

In order to determine the role of LdGDI, the BHb-loaded endosomes were treated with LdGDI or its mutant as described previously^20^. The endosomes were preincubated with fusion buffer containing protease inhibitors (1 mM phenyl methylsulfonyl fluoride, 20 ug/ml leupeptin and 20 ug/ml of aprotinin) for 20 min at 23 °C in the presence of 1 mM GDP. Subsequently, 6 μg/ml of LdGDI:WT or its mutant was added to the reaction mixture and incubated for 20 min at 23 °C. The endosomes were sedimented by centrifugation at 100,000 g for 5 min and the pellet was assayed for the presence of Rab proteins by western blot analysis using anti-LdRab5 antibody to determine the presence of Rab protein on the membrane after GDI treatment.

### *In vitro* reconstitution of endosome fusion in *Leishmania*

Reconstitution of fusion between endosomes prepared from *Leishmania* was carried out using a similar procedure described earlier^20^. Briefly, two sets of endosomes containing either BHb or AHRP were mixed in fusion buffer (250 mM sucrose, 0.5 mM EGTA, 20 mM HEPES-KOH, pH 7.2, 1 mM dithiothreitol, 1.5 mM MgCl_2_, 100 mM KCl, and 0.25 mg/ml avidin as scavenger; including an ATP regenerating system, 1 mM ATP, 8 mM creatine phosphate, 31 units/ml creatine phosphokinase) supplemented with gel filtered (G-25 Sephadex) cytosol prepared from *Leishmania.* Fusion was carried out for 1 h at 23 °C and the reaction was stopped by chilling on ice. The membrane was solubilized in solubilization buffer (SB, PBS containing 1% Triton X-100 and 0.2% methylbenzethonium chloride with 0.25 mg/ml avidin as scavenger). Finally, the BHb-AHRP complexes were immunoprecipitated using anti-Hb antibody and the HRP activity associated with BHb-AHRP complex was measured as fusion unit using O-phenylenediamine as the chromogenic substrate. Fusion carried out at 4 °C or without cytosol was low and was subtracted from the other values to determine specific fusion.

### Role of LdGDI in the extraction of Rab protein in *Leishmania*

To determine the role of LdGDI and its mutant in the extraction of Rab proteins from the respective intracellular membrane, *Leishmania* promastigotes overexpressing GFP-LdGDI:WT or GFP-LdGDI:R239A were transfected with RFP-LdRab1:WT, RFP-LdRab5:WT or RFP-LdRab7:WT construct using same protocol as described previously. Transfected cells were then incubated on ice for 10 min and transferred into G418 (30 μg/ml) containing M199 medium for 24 h at 23 °C. Subsequently, positive clones expressing both proteins were selected in the presence of G418 (30 μg/ml) and Blasticidin (15 μg/ml). Membrane and cytosolic localization of indicated RFP-LdRab proteins in GFP-LdGDI:WT or GFP-LdGDI:R239A over-expressing *Leishmania* were determined by confocal microscopy.

### Role of LdGDI in the trafficking of Hb in *Leishmania*

Intracellular trafficking of Hb in *Leishmania* overexpressing LdGDI:WT or its mutant was determined as described previously^21^. Briefly, cells (10^7^ cells/ml) were washed twice and resuspended in 250 μl of ice-cold vPBS containing Alexa Fluor-594 labelled Hb (120 μg/ml) and incubated at 23 °C for 5 min to label the early endosomal compartment. Cells were washed three times with cold vPBS to remove unbound Alexa-Hb and resuspended in prewarm (23 °C) vPBS for indicated periods of times. At respective times, cells were transferred onto ice, washed with chilled vPBS and visualized under a Zeiss LSM 510 META confocal microscope.

### Role of LdGDI in the secretion of gp63 in *Leishmania*

To determine the role of LdGDI in the secretory pathway of *Leishmania*, secretion of gp63 by LdGDI or its mutant overexpressing *Leishmania* in the growth medium was measured as described previously^27^. Briefly, indicated *Leishmania* (1 × 10^7^) were grown in 1 ml of FCS free M199 medium for 24 h at 23 °C. Subsequently, cells were pelleted by centrifugation (1500 g for 10 min at 4 °C) and supernatant from the respective culture was collected. Secreted proteins in resultant supernatant were acetone precipitated. The secretion of gp63 by indicated cells were analyzed by western blot using anti-gp63 antibody. Respective cell pellets were also analyzed by western blot using specific antibody.

## Additional Information

**How to cite this article**: Shanmugam, S. *et al*. Single GDP-dissociation Inhibitor Protein regulates endocytic and secretory pathways in *Leishmania. Sci. Rep.* **6**, 37058; doi: 10.1038/srep37058 (2016).

**Publisher's note:** Springer Nature remains neutral with regard to jurisdictional claims in published maps and institutional affiliations.

## Supplementary Material

Supplementary Information
