# Supplementary material for: Single GDP-dissociation Inhibitor Protein regulates endocytic and secretory pathways in Leishmania
Source: Sci Rep. 2016 Nov 14;6:37058. doi: 10.1038/srep37058 (PMC5107955; doi:10.1038/srep37058)

**Single GDP-dissociation Inhibitor Protein regulates endocytic and secretory pathways in *Leishmania*.**

**Senthil kumar Shanmugam#, Kamal Kumar#, Pawan Kishor Singh, Ruchir Rastogi and Amitabha Mukhopadhyay¶**

**National Institute of Immunology, Aruna Asaf Ali Marg, New Delhi 110067, India.**

**Supplementary Figure 1.**

**Expression and purification of recombinant LdGDI protein**

To purify GDI as a GST fusion protein, *Escherichia coli* (BL21 strain) containing respective constructs were grown to an $O.D._{600}$ of 0.5 in LB. Cells were treated with 0.2 mM isopropyl 1-thio-β-D-galactopyranoside (IPTG) for 3 h at 30 °C to induce the expression of GST-fusion protein, and the respective fusion proteins were affinity-purified as per the manufacturer's instructions (Amersham Biosciences) using glutathione-Sepharose 4B. Briefly, bacterial cells were harvested by centrifugation and cells were lysed as described previously[27]. Finally, proteins from cell lysate were extracted with Triton X-100 (1%) and cell debris was separated by centrifugation at 18500 g for 10 min at 4°C. The resultant supernatant was incubated with glutathione-Sepharose beads for 1 h at 4°C. Beads were extensively washed with PBS, recombinant proteins were eluted from the beads in 50 mM Tris-HCl containing 30 mM glutathione, pH 9.0, and dialyzed against PBS. Purity of the proteins was checked by SDS-PAGE.

Our results showed the purification of expected size (75 kDa) of GST-LdGDI to homogeneity (lane 4). Lane1, Protein marker; Lane 2, Uninduced lysate of *E. coli*; Lane3, Induced lysate of *E.coli*; Lane4, Purified GST-LdGDI. Similarly, cells transformed with mutant plasmid were used to purify the GST-LdGDI:R239A fusion protein.

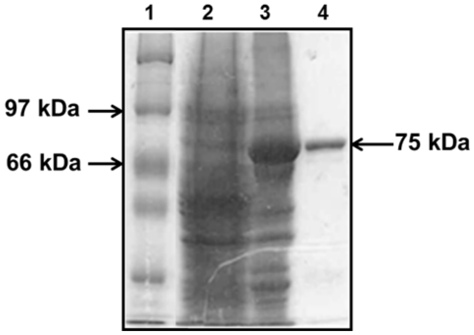

**Supplementary Figure 2.**

Un-cropped images showing the localization of LdGDI:WT and its mutant in *Leishmania*. Full length blot showing the levels of overexpression of LdGDI:WT and its mutant as GFP fusion proteins in *Leishmania*.

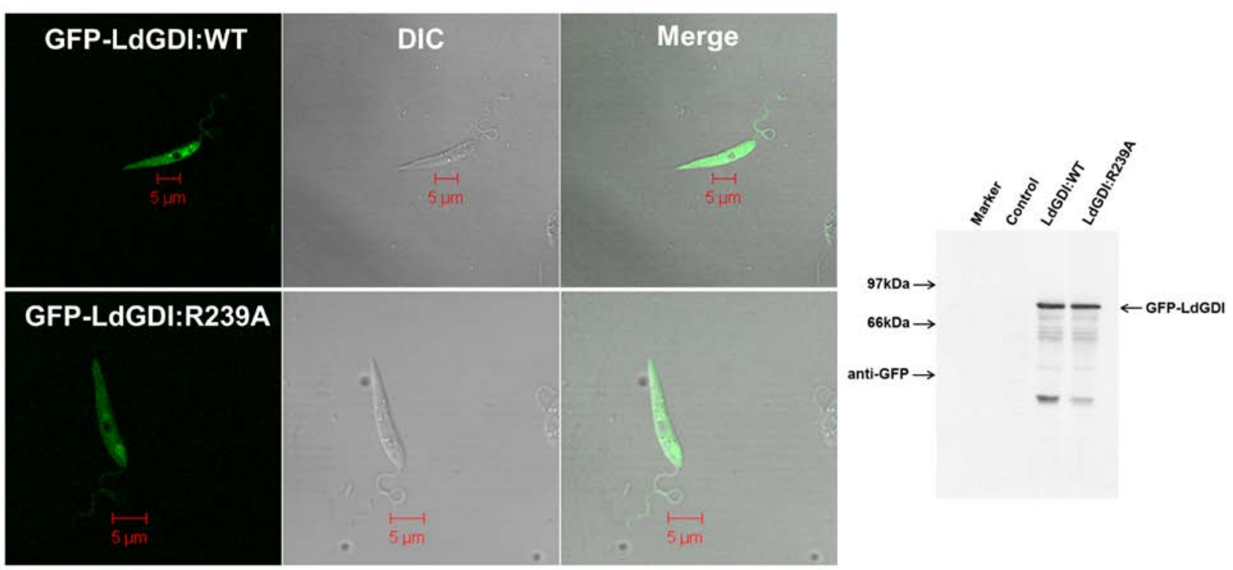


**Supplementary Figure 3.**

Full length blot showing the extraction of LdRab5 from the endosome by LdGDI. Endosome were treated with LdGDI:WT or mutant in the presence of GDP. Treated endosomes were centrifuged; pellets (P) and supernatant (S) were analyzed for the presence of LdRab5 by western blots using anti-LdRab5 antibody.

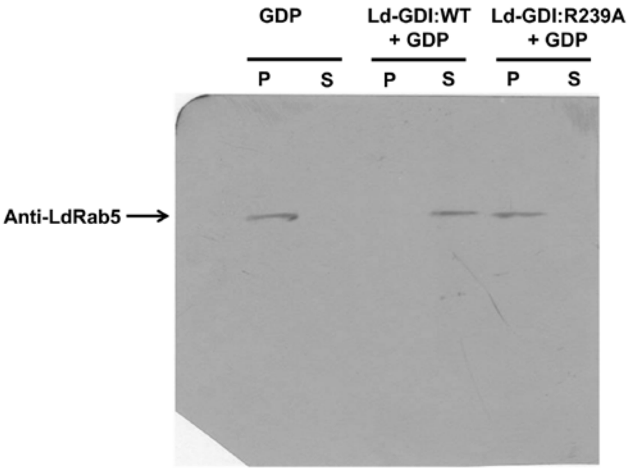

**Supplementary Figure 4.**

Full length blot showing the role of LdGDI in the secretory pathway in *Leishmania*. To determine the role of LdGDI in the secretory pathway in *Leishmania*, we determined the levels of cell-associated and secreted gp63 by respective *L. donovani* promastigotes by western blot analysis using specific antibodies.

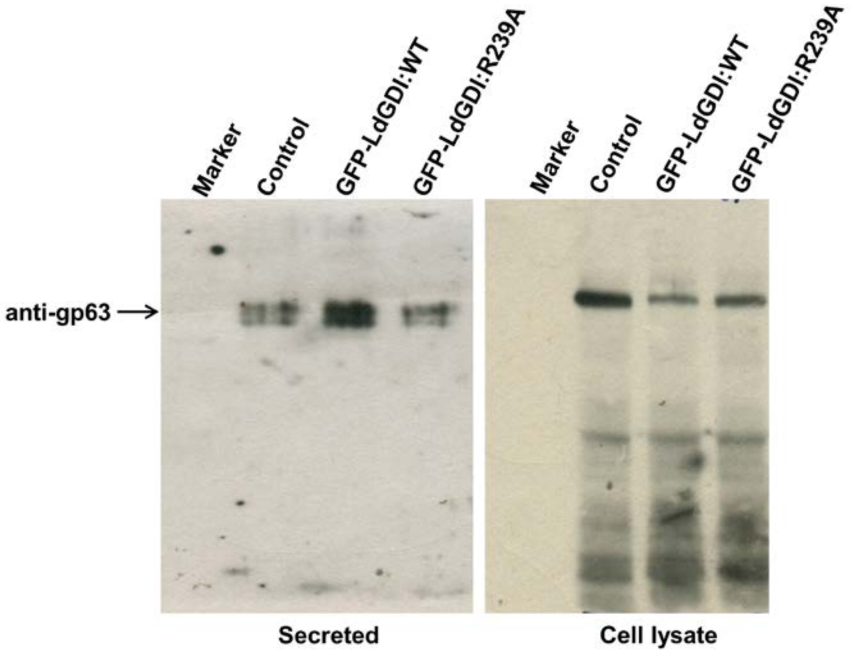

Supplement: Supplementary Information [file srep37058-s1.pdf]
